# Effective Communication to Prevent Infectious Disease in Women^1^

**DOI:** 10.3201/eid1011.040623_13

**Published:** 2004-11

**Authors:** Blanca Torres, Meredith Hickson, Marin P. Allen, Aida L. Giachello, Catalina R. Ramos-Hernandez, Yolanda Partida

**Affiliations:** * Centers for Disease Control and Prevention, Atlanta, Georgia, USA;; † National Institutes of Health, Bethesda, Maryland, USA;; ‡ University of Illinois at Chicago, Chicago, Illinois, USA;; § University of Southern California, Los Angeles, California, USA

**Keywords:** communication, infectious disease, women, literacy, diversity, latino

## Health Literacy Within a Cultural Context

According to Healthy People 2010, health literacy is "the degree to which individuals have the capacity to obtain, process, and understand basic health information and services for appropriate health decisions." Health literacy incorporates a range of abilities, including reading, comprehending, and analyzing information; decoding instructions, symbols, charts, and diagrams; weighing risks and benefits; and ultimately, making decisions and taking action. Areas commonly associated with health literacy concerns are patient-physician communication, drug labeling, medical instructions and medical compliance, health information publications and other resources, informed consent, responding to forms, giving patient history, public health training, and assessments for allied health programs. Cultural competence is the body of knowledge, belief, and behavior that needs to be understood "not as an appliqué, but as part of the fabric" of communication with different populations. Cultural competence, as expressed by the National Center for Cultural Competence, includes the institutionalization of cultural knowledge and the evolution of competence over time.

## Cultural Diversity and Institutional Inequalities

Key concepts are cultural knowledge, cultural sensitivity, cultural diversity, and cultural competency. Strategies exist to develop linguistically, culturally, educationally, and gender-appropriate services at the individual and institutional levels. Demographic changes in the nation's populations, the history of social activism, and health disparities are all factors related to institutional racism, sexism, and social discrimination. A template can be used to develop a strategy to assess an organization's level of cultural competency and to develop an overall plan to address the concerns identified.

## Improving Patient-Provider Communication for Latinos

Ten demonstration sites across the country have been established to develop practical, affordable solutions that health organizations can use to eliminate language barriers and improve the quality of care for Latino patients. These programs are improving the availability and quality of interpreters and developing materials in Spanish. By using the first valid and reliable tools to be developed, demonstration sites can assess language proficiency and interpreting skills of persons used as interpreters. Challenges and barriers to translating English documents to Spanish need to be addressed.

Using untrained interpreters presents some risks, and no standards and training for health interpreters currently exist. Interpreters must be trained, certified, available, and integrated into the healthcare system. A new, universal set of healthcare signage is based on symbols. Regional language differences and the evolution of language are concerns in a multicultural environment. Cultural processes are important to language development, and emphasis should be placed on the need to improve understanding in patient-provider communication.

